# Evaluation of Asymptomatic Malaria Status in Eastern of Afghanistan Using High Resolution Melting Analysis

**Published:** 2020

**Authors:** Sayed Hussain MOSAWI, Abdolhossein DALIMI, Najibullah SAFI, Fatemeh GHAFFARIFAR, Javid SADRAEI

**Affiliations:** 1. Department of Medical Parasitology, Faculty of Medical Sciences, Tarbiat Modares University, Tehran, Iran; 2. World Health Organization Country Office, Kabul, Afghanistan

**Keywords:** Asymptomatic malaria, Afghanistan, High resolution melting analysis

## Abstract

**Background:**

Malaria is threatening more than half of Afghanistan population. Asymptomatic malaria is notable problem against malaria controlling strategies. In this study we evaluated the asymptomatic malaria status in Nangarhar Province, Afghanistan in 2017.

**Methods:**

Overall, 296 finger blood samples were taken on DNA Banking Cards and microscopic slides from asymptomatic individuals in Jalalabad city. We used a novel post real time PCR high resolution melting analysis beside microscopy and semi-nested multiplex PCR to evaluate status of asymptomatic malaria in this city.

**Results:**

The prevalence of asymptomatic malaria in Jalalabad city was determined 1.7% (5/296), 7.43% (22/296) and 7.78% (26/296) by microscopy, Seminested multiplex PCR and qRT-PCR-HRM, respectively. Out of 26 positive cases were detected by qRT-PCR-HRM, 21, 1 and 4 cases were detected *P. falciparum*, *P. vivax* and mixed infection of *P. falciparum* and *P. vivax*, respectively.

**Conclusion:**

Our data indicating on existence of significant number of asymptomatic reservoirs that assists in prolonged endemicity of the disease. On the other hand, the molecular methods are better alternatives for microscopy especially for monitoring of asymptomatic cases of malaria.

## Introduction

Five species of *Plasmodium* genus (i.e., *Plasmodium P. falciparum*, *P. vivax*, *P. ovale*, *P. malariae*, and *P. knowlesi*) are causative agents of human malaria. Despite many efforts towards eliminating malaria, there were 212 million new cases and 429000 deaths in 2015. Malaria had always been one of public health complications in Afghanistan. More than half of the population live in malaria-endemic regions. *Plasmodium vivax* is the dominant species (70%–95%) and *P. falciparum* located in the second place (5%–30%) (1–3).

Malaria severity varies from symptomatic (complicated, mild and uncomplicated), to asymptomatic. Symptomatic malaria has been at the core of epidemiological studies' attention thanks to its mortality and morbidity. The manifestations of complicated malaria encompass cerebral malaria, convulsions, malarial anemia, haemoglobinuria, hypoglycemia, metabolic acidosis (associated with respiratory distress), acute pulmonary edema, acute renal failure, jaundice, circulatory collapse, hyperparasitaemia, high fever electrolyte disturbance, and/or spontaneous bleeding. On the other hand mild or uncomplicated malaria offers manifestations like fever, chills and sweats, headache, vomiting, watery diarrhea, anemia, jaundice, and splenomegaly (4, 5).

Actually, the aforementioned symptoms have not been seen in asymptomatic carriers. A carrier for the *Plasmodium* parasite of any density who displays no fever or other acute symptoms and have not received recent antimalarial drugs is called asymptomatic. Therefore, asymptomatic carriers play a role as a reservoir that leading to persistence of malaria transmission within their localized populations (6, 7).

Although conventional methods like microscopy have been considered as the “gold standard”, it possesses low sensitivity for malaria diagnosis (100 to 200 parasites/μl of blood). However, the molecular approaches like nested PCR and real-time PCR offer detection of *plasmodium* spp. at lower concentration: 5 parasites/μl and down to 0.02 parasite/μl respectively. High-resolution melting (HRM) analysis is a cost-benefit implement for post-real-time analysis of PCR products. It does not require post PCR manipulating and detect multiplex species simultaneously (8, 9).

There are some works carried out in Afghanistan especially on drug resistance evaluation, risk prediction, vector controlling and epidemiological studies (10–12).

Unfortunately there has been no attempt to evaluate the asymptomatic malaria status in Afghanistan so far. In this study we were planning to evaluate the asymptomatic malaria status in Nangarhar province by make a use of real-time PCR-HRM.

## Materials and Methods

### Study of the location and sampling

A cross-sectional study was performed by available sampling method in 2017. In this study, referred individuals to the Nasiri laboratory were checked whether they have been suffered malaria since at least 6 months ago or possessing any symptoms of malaria, especially body temperature. These patients had issues other than malaria and febrile individuals were omitted from the study after checking by a physician. Out of them, only 296 individuals were selected as not malaria affected individuals. Then 296 finger blood samples were taken on DNA Banking Cards (Kowsar Biotechnology Center, Tehran, Iran) and microscopic slides. Nangarhar province (34.4198° N, 70.4729° E) is one of the hyper malaria endemic regions that located in the East of Afghanistan.

### Ethical approval

Approval of ethics application with appropriate experimental protocols was taken from Medical Ethic Committee of Tarbiat Modares University, Tehran, Iran. All the protocols used in this study were in accordance with the approved guidelines (IR.TMU.REC.1395.401) and informed agreement were taken from all people participated in this study.

### Microscopic analysis

Microscopic analysis was performed on prepared thick and thin blood films under ×100 lens with oil immersion. Totally 200 good fields were examined for detection of parasites and a double check procedure was performed for identification of mixed cases. For quality control 10% of negative cases and all of positive cases were double-checked bye a skilled microscopist. For final confirmation, the slides and their pictures were sent to the National Malaria Center, Tehran University of Medical Sciences, Tehran, Iran.

### Molecular analysis

#### DNA extraction

DNA extraction was performed on DNA banking cards (DBCs). After punching 2 mm discs from the DBCs, 3–4 discs were applied for DNA extraction by the SinaPure DNA extraction kite (CinnaGen Tehran, Iran). We added 3-4 discs to a sterile 1.5 or 2 polypropylene tube then add 400 μl Lysis buffer and vortex at max speed for 1 min. In the next step we added 300 μl Precipitation solution and vortex at max speed for 30 sec. The solution was transferred to a spin column with collection tube by pipetting and centrifuged at (12.100×g) for 1 min. The collection tube was discarded. Column spin were placed in new collection tube then 400 μl Wash buffer I was added and centrifuged at 12.100×g for 1 min. After discarding the flow-through. The spin-column Washed with 400 μl of Wash buffer II twice at centrifugation of 12.100×g for 1 min. The flow-through was discarded and the column carefully transferred to a new 1.5 ml tube. 30 μl 65 °C preheated elution buffer or TE buffer was placed in the center of the column, lid closed and incubate for 3–5 min at 65 °C. Thereafter, we centrifuged at 12.100×g for 1 min and eluted DNA were kept in −20.

#### Semi nested multiplex PCR

Two rounds of semi-nested PCR (SNM-PCR) were performed (13). Briefly in first round UNR and PLF primers were applied for amplification of about 783–821 bp fragment of 18S SSrRNA gene of *Plasmodium* species. In the second round the PLF primer, 2 μl of diluted product of first round and four primers of *plasmodium* spp. (VIR, FAR, MAR and OVR) were added into 12.5 μl of “2X Taq Master Mix ReD” (Amplicon inc., containing 150 mM Tris-Cl PH 8.5, 40 mM (nh4)2So4, 3 mM Mgcl2, 0.2% tween 20, 0.4 mM dntPs, 0.05 U/μl Taq DNA polymerase, inert red dye and stabilizer), to reach final concentration of 25 μl. The products of the second round (499 bp for *P. vivax* and 395 bp for *P. falciparum*) were seen in 2% agarose gels.

#### Real-time polymerase chain reaction coupled with high-resolution melting analysis (real-time PCR-HRM)

The high resolution melting analysis was performed on 18S SSrRNA gene for detection of *Plasmodium* species as described by Chua (8).

**Plasmid DNA construction:** Briefly, the amplification of 18S SSrRNA gene was performed on two clinical samples confirmed by microscopy (one *P. falciparum* and one *P. vivax*). Then the pTG19-T vector was employed to clone the amplicones directly through TA cloning technique (PCR TA Cloning kit, CinnaClon Co., Iran). The colony PCR was performed by make use of universal primers; M13F: AGGGTTTTCCCAGTCACGA and M13R: GAGCGGATAACAATTTCACAC for confirmation of the cloning (14). Finally, the extracted plasmids have been sent for sequencing and the results have been deposited in the GenBank database under accession numbers: MG518389 and MG518390.

The real-time PCR-HRM assay was performed using an ABI 7500 Fast Real-time PCR system (Applied Biosystems, Inc.). For amplification, 0.1 μM of each primers (forward primer 5′-GRAACTSSSAACGGCTCATT-3′ and reverse primer 5′ -ACTCGATTGATACACACTA-3′), 4μl (total 20 μl) of extracted DNA and 4 μl of 5X Hot Firepol® EvaGreen® HRM Mix (Solis BioDyne, Tartu, Estonia) were used in qPCR 8-strip tubes (Gunster Biotech, Taiwan).

The thermal program included an initial denaturation at 95 °C for 15 min, followed by 40 cycles of amplification consisting of 95 °C for 15 sec (denaturation step), and 60 °C for 1 min (primer annealing and elongation step). The amplicons were then subject to a melt program raised from 60 °C to 95 °C. During HRM, the amplicons were denatured before development of melting curves in the inflexion point, where changes in fluorescence concerning changes in temperature (dF/dT) were recorded with a ramp of 0.3 °C/sec. Ultimately, obtained melting curve profiles were analyzed by making a use of HRM software for Windows ® version 3.0.1. (Applied Biosystems).

### Phylogenetic analysis

The PCR product of several samples and primers were sequenced by the ABI3730XL sequence analyzer (Macrogen, Korea). Editing and aligning of sequences with reference sequences from GenBank, were performed by using two commercial software packages (Sequencher v. 4.4 and CLCMainWorkbench5.5). The phylogenetic tree was created for *P. falciparum* and *P. vivax* 18S rRNA sequences with the neighbor-joining algorithm using Molecular Evolutionary Genetics Analysis (MEGA) software, version 7.0 (15).

### Statistical analysis

For analysis of the data in the present work, the chi-squared test performed by making use of software package SPSS, ver. 14.0. In addition, this software package was used for determining and comparing of the Sensitivity and Specificity among different diagnosis methods. The significance level was considered by 95% confidence interval.

## Results

### Demographic characteristics of participants

Among 296 participants, there were 175 (59.1%) male and 121 (41.9%) female with a mean age of 23 ((IQR: 5–71 and SD: 11.02). The statistical analysis showed that there were no significant association between sex (*P*=0.631) and age (*P*=0.694) in taking asymptomatic malaria.

### Comparison of microscopic and molecular methods in detection of asymptomatic infections

**Fig. 1:**
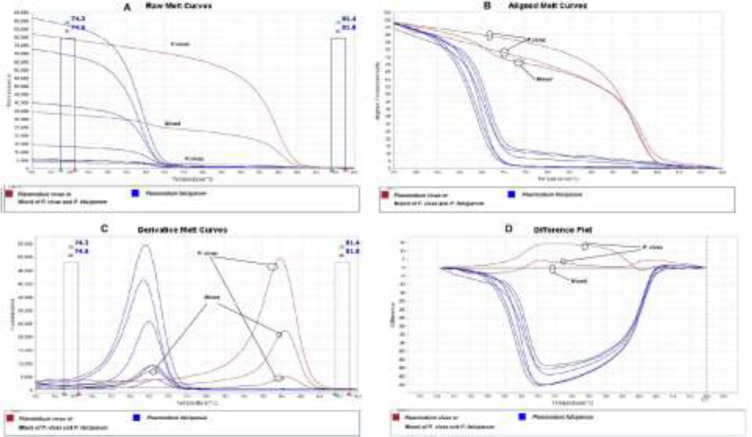
Melting curve variance of asymptomatic malaria in (A) raw, (B) aligned (C) derivative and (D) difference plot analyses. The melting curves and Tm for each species and mixed infections can be very well understand

**Fig. 2:**
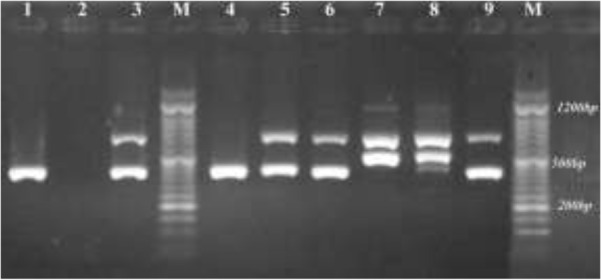
SNM-PCR analysis of 18S of SS r RNA gene on gel electrophoresis. Lane1: positive control of *P. falciparum*, Lane2: negative control, Lanes 3, 4, 5, 6, and 9: samples recognized as *P. falciparum*; Lane7: sample recognized as *P. vivax* and Lane 8 sample recognized as mixed infection of *P. vivax* and *P. falciparum*. M: 50 bp marker

In general, 1.7%, 7.43% and 8.78% of the samples were detected positive for malaria by microscopy (two *P. vivax* and three *P. falciparum*), SNM-PCR (one *P. vivax*, twenty *P. falciparum* and one mixed) and real-time PCR-HRM (one *P. vivax*, twenty-one *P. falciparum* and four mixed), respectively (Table 1). The results of melting curve variance of real-time PCR-HRM are shown in Fig. 1 and the amplified 18S SSrRNA gene fragments on gel electrophoresis are shown in Fig. 2. Finally, the prevalence of asymptomatic malaria was calculated at 8.9%.

**Table 1:** Detection of asymptomatic malaria by different methods in the patients with different age and gender in Jalalabad city, Afghanistan

***Methods***	***Detection***		***Gender***			***Age (Year)***					***Detection of asymptomatic malaria***	
			***Male***	***Female***	***Total***	***<=10***	***11–20***	***21–30***	***>=31***	***Total***		
HRM	*P. falciparum*	Count	13	8	21	3	8	6	4	21	21	8.78%
		% within HRM	61.9%	38.1%	100.0%	14.3%	38.1%	28.6%	19.0%	100.0%	7.1%	
	*P. vivax*	Count	1	0	1	0	0	1	0	1	1	
		% within HRM	100.0%	0.0%	100.0%	0.0%	0.0%	100.0%	0.0%	100.0%	0.33%	
	Mixed	Count	4	0	4	0	3	1	0	4	4	
		% within HRM	100.0%	0.0%	100.0%	0.0%	75.0%	25.0%	0.0%	100.0%	1.35%	
	Negative	Count	157	113	270	23	105	70	72	270	270	
		% within HRM	58.1%	41.9%	100.0%	8.5%	38.9%	25.9%	26.7%	100.0%	91.21%	
	Total	Count	175	121	296	26	116	78	76	296	296	
		%	59.1%	40.9%	100.0%	8.7%	39.2%	26.3%	25.7%	100.0%	100.0%	
SNM-PCR	*P. falciparum*	Count	13	7	20	3	8	6	3	20	20	7.43%
		% within Sn m-PCR	65%	35%	100.0%	15%	40%	30%	15%	100.0%	6.7%	
	*P. vivax*	Count	1	0	1	0	0	1	0	1	1	
		% within Sn m-PCR	100.0%	0.0%	100.0%	0.0%	0.0%	100.0%	0.0%	100.0%	0.33%	
	Mixed	Count	1	0	1	0	1	0	0	1	1	
		% within Sn m-PCR	100.0%	0.0%	100.0%	0.0%	100.0%	0.0%	0.0%	100.0%	0.33%	
	Negative	Count	160	114	274	23	107	71	73	274	274	
		% within Sn m-PCR	58.4%	41.6%	100.0%	8.4%	39.1%	25.9%	26.6%	100.0%	92.5%	
	Total	Count	175	121	296	26	116	78	76	296	296	
		%	59.1%	40.9%	100.0%	8.7%	39.2%	26.3%	25.7%	100.0%	100.0%	
Microscopy	*P. falciparum*	Count	1	2	3	0	1	1	1	3	3	1.7%
		% within Microscopy	33.3%	66.7%	100.0%	0.0%	33.3%	33.3%	33.3%	100.0%	1.01%	
	*P. vivax*	Count	2	0	2	0	0	2	0	2	2	
		% within Microscopy	100.0%	0.0%	100.0%	0.0%	0.0%	100.0%	0.0%	100.0%	0.67%	
	Mixed	Count	0	0	0	0	0	0	0	0	0	
		% within Microscopy	0%	0%	0%	0%	0%	0%	0%	0%	0%	
	Negative	Count	172	119	291	26	114	76	75	291	291	
		% within Microscopy	59.1%	40.9%	100.0%	8.9%	39.2%	26.1%	25.8%	100.0%	98.3%	
	Total	Count	175	121	296	26	116	78	76	296	296	
		%	59.1%	40.9%	100.0%	8.7%	39.2%	26.3%	25.7%	100.0%	100.0%	

The performance of molecular tests was assessed using microscopy as the gold standard. Real-time PCR-HRM has higher sensitivity (100% with 47.8% to 100% confident internal) and specificity (92.7% with 89.1% to 95.4% confident internal); besides, the positive predictive values (PPV) and negative predictive values (NPV) were 100% and 92.7%, respectively (Table 2). On the other hand, SNM-PCR showed the same sensitivity, and no statistical significant difference (*P*=0.883) observed in specificity (91.1% with 81.1% to 94.7% confident internal). Comparison of results for microscopy with three real-time PCR based methods that have been used in this study and two others is shown in Table 3.

**Table 2:** Comparison of semi nested mulitiplex PCR and qRT-PCR-HRM methods with the microscopy as gold standard in detecting asymptomatic malaria in Jalalabad City, Afghanistan

***Variable***	***Reference***		***Total***	***Validity***	
SNM-PCR	Microscopy			Sensitivity (95%Cl)	Specificity (95%Cl)
	Positive	Negative		100% (47.8 – 100)	91.1% (86.1 – 94.7)
Positive	5	17	22		
Negative	0	274	274		
Total	5	291	296		
Qrt-PCR-HRM	Positive	Negative		Sensitivity (95%Cl)	Specificity (95%Cl)
Positive	5	21	26	100% (47.8 – 100)	92.7% (89.1 – 95.4)
Negative	0	270	270		
Total	5	291	296		

**Table 3:** The comparison of microscopy with three real-time PCR based methods that have been used in this study and two other studies

***Variable***	***Result***	***Microscopy***			***Sensitivity (%) (95%Cl)***	***Specificity (%) (95%Cl)***	***PPV (%)***	***NPV (%)***	***KC (%)***	***Accuracy***	***DOR***	***Disease prevalence (%)***
		***Positive***	***Negative***	***Total***								
The present study (qRT-PCR-HRM)	Positive	5	21	26	100 (47.8–100)	92.7 (89.1–95.4)	100	92.7	30.2	0.929	1.02	8.78
	Negative	0	270	270								
	Total	5	291	296								
Zhao et al. 2017 (nRT-PCR)	Positive	12	175	187	100 (73.5–100)	82.3 (79.8–84.7)	6.4	100	10	0.82	1.19	1.19%
	Negative	0	818	818								
	Total	12	993	1005								
Wang et al. 2014 (Real-time PCR)	Positive	14	39	53	93.3 (68–99.8)	97.4 (96.5–98.1)	26	99	40.2	0.97	1.05	0.97
	Negative	1	1498	1499								
	Total	15	1537	1552								

PPV: positive predictive values, NPV: negative predictive values, DOR: Diagnostic odd ratio and KC: Kappa Coefficient

**Fig. 3:**
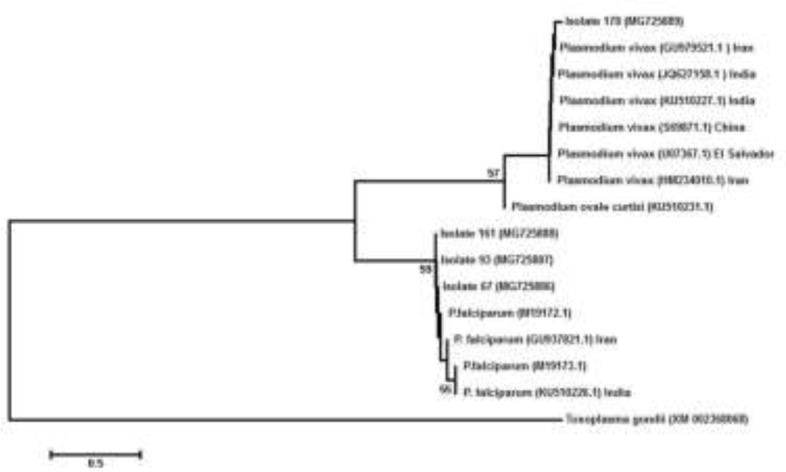
Phylogenetic relationships of *Plasmodium* species of 18S ribosomal RNA gene of the collected samples from Jalalabad, Afghanistan (accession numbers MG725886 –MG725889), compared with other species in GenBank by the neighbour-joining algorithm

The phylogenetic links of *Plasmodium* species from Jalalabad city were compared with other species in GenBank using the neighbor-joining algorithm (Fig. 3). The obtained sequences of this study have high degree of homology to the isolates from other countries. The sequences obtained were annotated in GenBank by accession numbers from MG725886-MG725889.

## Discussion

Malaria is endemic in Afghanistan that threaten about half of the Afghan population. According to the annual reports of malaria cases from the ministry of public health, the disease cases have been increased especially in the eastern regions in recent years. In this preliminary study, we aimed to show the frequency of asymptomatic malaria in Nangarhar province by one of newest diagnosis approaches. Asymptomatic carriers play a significant role as reservoirs of *Plasmodium* spp. Therefore, they are potential and a dormant risk for maintenance of the disease in endemic area. Afghanistan is a country in a controlling phase of malaria and exploration of asymptomatic individuals could help in better performance of this task (16).

There are different limit of detection between microscopic, conversional and qRT-PCR. There are many problems in front of microcopy, a method that is common in developing countries like Afghanistan. These problems include low sensitivity, human errors, even in well trained personnel, time-consuming, false-positive and bad condition of smear preparation. In some studies, the significant sensitivity, specificity, and accuracy of molecular methods have been proved, especially in detection of samples with low parasitemia like asymptomatic individuals. In low parasitemia samples the amount of gene copy number is vital, so in many studies, SSU rRNA gene have been applied for detection *Plasmodium* spp. In this study, we compared the microscopy with SNM-PCR and real-time PCR–HRM for detection of asymptomatic malaria. The two molecular methods are more sensitive and accurate than microscopy. However, since different problems include: post PCR manipulation, lowest sensitivity in detection of mixed infection, and time-consuming change the situation in favor of real-time PCR–HRM (17–23).

Real-time PCR-HRM have been used for detection and genotyping of parasitic diseases like echinococcosis, leishmaniasis, fascioliasis and malaria. This method used for detection of low *Plasmodium* parasitaemia infections among microscopically negative febrile patients in Kenya (23–25).

With a comparison among this work and two other real-time-PCR based researches, acceptable sensitivity[Zhao, 2017 #1], specificity, accuracy and diagnostic odds ratio (>1) were seen in all of these techniques. Compared with these real-time PCR-based methods, microscopy showed unremarkable agreement, with kappa values. This is while real-time PCR-HRM showed more powerful performance in detection of true positives and true negatives (21, 26).

There are many studies on asymptomatic malaria all over the world. Iran located in the west of Afghanistan is a country in the elimination phase. Studies in this country were done mostly in the southern regions and indicating on the absence of asymptomatic carriers. Although there are no data about asymptomatic malaria in Pakistan (south and east of Afghanistan), there are some works in India highlighted the existence of high prevalence of the reservoirs, especially in eastern populations. In Africa, there is a high prevalence of the asymptomatic malaria in African countries (27–33).

In this study, the majority form of asymptomatic malaria in Nangarhar province is *P. falciparum* (78.8 %) that is a warning for National Malaria and Leishmaniasis Control Program. Meanwhile, while our study was conducted, the most cases of malaria were *P. vivax* and rarely *P. falciparum* were observed in Jalalabad. This hidden malaria will assist in persist of severe form of malaria in the region, so it is worth doing an active case detection of asymptomatic infections (34).

This study is the first attempt for evaluation of asymptomatic malaria in endemic regions in Afghanistan. This investigation could be promoted by performing on largest sample volumes and in a vast endemic area of Afghanistan. The insufficient security and facilities (like round clock electricity) are big problems in front of establishing molecular methods in Afghanistan. Therefore, for promoting the malaria control program capacities the improvement of infrastructures is essential.

## Conclusion

*P. falciparum* is the common form of asymptomatic malaria in Nangarhar province. This should be taken into consideration by malaria managers, however, the existence of mixed infection and *P. vivax* should not be neglected. On the other hand, the molecular methods are better alternatives for microscopy especially for monitoring of asymptomatic cases of malaria.
